# EEG connectivity and network analyses predict outcome in patients with disorders of consciousness – A systematic review and meta-analysis

**DOI:** 10.1016/j.heliyon.2024.e31277

**Published:** 2024-05-15

**Authors:** Danuta Szirmai, Arashk Zabihi, Tamás Kói, Péter Hegyi, Alexander Schulze Wenning, Marie Anne Engh, Zsolt Molnár, Gábor Csukly, András Attila Horváth

**Affiliations:** a Centre for Translational Medicine, Semmelweis University, Budapest, Hungary (Baross utca 22., Budapest, H-1085, Hungary; b Neurocognitive Research Center, National Institute of Mental Health, Neurology, Neurosurgery, Budapest, Hungary (Amerikai út 57., Budapest, H-1145, Hungary; c Mathematical Institute, Department of Stochastics, Budapest University of Technology and Economics, Budapest, Hungary (Műegyetem rkp. 3, Budapest, H-1111, Hungary; d Department of Anatomy, Histology and Embryology, Semmelweis University, Budapest, Hungary (Üllői út 26., Budapest, H-1085, Hungary; e Institute of Pancreatic Diseases, Semmelweis University, Budapest, Hungary (Tömő u. 25-29, Budapest, H-1083, Hungary; f Institute for Translational Medicine, Medical School, University of Pécs, Pécs, Hungary (Szigeti út 12., Pécs, H-7624, Hungary; g Department of Anesthesiology and Intensive Therapy, Semmelweis University, Budapest, Hungary (Üllői út 78., Budapest, H-1082, Hungary; h Department of Anesthesiology and Intensive Therapy, Poznan University of Medical Sciences, Poznan, Poland (49 Przybyszewskiego St, Poznan, Poland, 60-355, Poland; i Department of Psychiatry and Psychotherapy, Semmelweis University, Budapest, Hungary (Balassa u. 6, Budapest, H-1083, Hungary

**Keywords:** DOC, Disorders of consciousness, Outcome prediction, EEG, CRS-R, Behavioural scale

## Abstract

Outcome prediction in prolonged disorders of consciousness (DOC) remains challenging. This can result in either inappropriate withdrawal of treatment or unnecessary prolongation of treatment. Electroencephalography (EEG) is a cheap, portable, and non-invasive device with various opportunities for complex signal analysis. Computational EEG measures, such as EEG connectivity and network metrics, might be ideal candidates for the investigation of DOC, but their capacity in prognostication is still undisclosed. We conducted a meta-analysis aiming to compare the prognostic power of the widely used clinical scale, Coma Recovery Scale-Revised - CRS-R and EEG connectivity and network metrics. We found that the prognostic power of the CRS-R scale was moderate (AUC: 0.67 (0.60–0.75)), but EEG connectivity and network metrics predicted outcome with significantly (p = 0.0071) higher accuracy (AUC:0.78 (0.70–0.86)). We also estimated the prognostic capacity of EEG spectral power, which was not significantly (p = 0.3943) inferior to that of the EEG connectivity and graph-theory measures (AUC:0.75 (0.70–0.80)). Multivariate automated outcome prediction tools seemed to outperform clinical and EEG markers.

## Highlights

• EEG connectivity markers and network metrics predict outcome in patients with prolonged disorders of consciousness.
• EEG spectral power measures do not perform significantly worse in outcome prediction, than connectivity and network metrics.
• Clinical behavioural scales (Coma Recovery Scale-Revised) have moderate capacity for outcome prediction.

## 1 Introduction

**Fig. 1:**
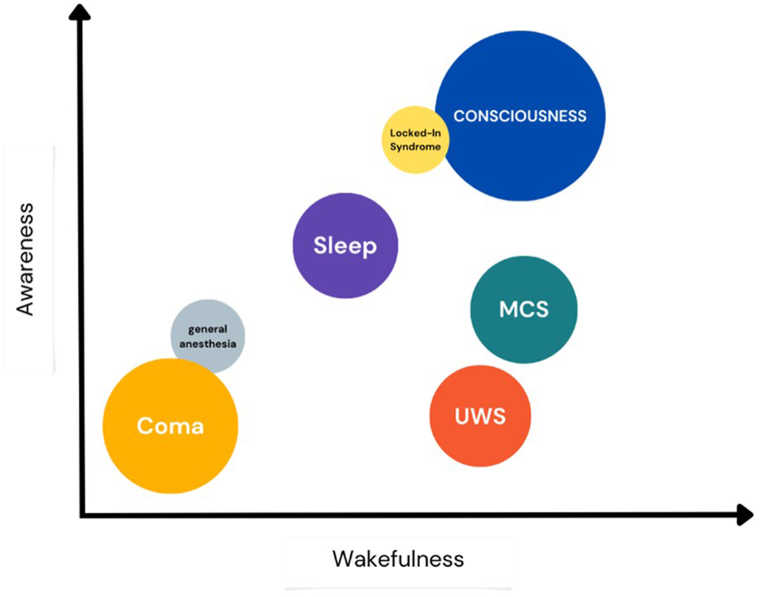
The continuum of consciousness (own graphic based on the article by Laureys et al., 2005).

Unresponsive wakefulness syndrome (UWS, previously known as ‘vegetative state’ (VS)) and minimally conscious state (MCS) characterized by ‘unconscious wakefulness’ are transitional states on the continuum from coma to wakefulness (Fig. 1). UWS patients do not exhibit any signs of purposeful behaviour or cognitive function, while MCS patients show some signs of purposeful behaviour, such as visual pursuit, fixation, or object manipulation [1,2]. If these states last longer than a month, they are considered prolonged disorders of consciousness (DOC).

The estimated prevalence of DOC ranges between 0.2 and 6.1/100 000 [3,4]. With advancing healthcare, DOC patients survive longer, posing ethical and economic burdens on doctors and caretakers.

Late recovery occurs in 10–24 % of cases, with some patients regaining consciousness even one year after the brain injury [5]. Uncertain estimation of the outcome of DOC patients might result in the withholding of treatment, which is the primary reason for death in this patient population [6].

Observational studies have highlighted that age [7], time since injury, etiology of DOC, and Coma Recovery Scale- Revised (CRS-R) total score are all good outcome predictors [8]. CRS-R is a frequently used standardized behavioural scale to assess the neurobehavioral status of patients. It is composed of 23 items, organized into 6 subscales that evaluate auditory, visual, motor, oromotor, communication, and arousal functions [9]. It is a sensitive tool for detecting conscious behaviour [10]. Previous reports highlighted that CRS-R performs better in differentiating UWS and MCS in acute and prolonged DOC than other scales like Full Outline of Unresponsiveness Score (FOUR) or Glasgow Coma Scale (GCS) [11,12], but similarly to other behavioural scales, it is dependent on patients’ attentiveness.

Better outcome prediction can be achieved with instrumental measures such as electroencephalography (EEG). Quantitative EEG can discriminate UWS from MCS, achieving high sensitivity and specificity [13]. EEG functional connectivity and network analysis perform better than spectral EEG measures in describing neurological conditions [14,15], and more generally, in describing higher order brain function [16,17].

International guidelines have not incorporated quantitative EEG for the prognostic evaluation of DOC[18,19], despite evidence supporting its good prognostic capacity. Previous meta-analyses assessed prognostic power of resting state EEG studies in DOC patients [20], and showed outstanding results; however, they did not provide quantitative results about the predictive capacity of EEG functional connectivity measures and network metrics.

We aimed to compare the prognostic power of the widely used CRS-R scale and EEG functional connectivity and graph-theory measures in patients with prolonged DOC.

## 2 Methods

### 2.1 Searching method

We followed the Cochrane Collaborations recommendations and we reported the meta-analysis in accordance with the PRISMA guidelines. We registered the review on PROSPERO (ID: CRD42022368745). We conducted a systematic search in 3 databases (PubMed, Embase, Cochrane). The search was initiated on 16 of November 2022 with the following search key: EEG AND (connectivity OR network OR phase OR amplitude OR coherence) AND (coma OR DOC OR "disorders of consciousness" OR consciousness OR conscious* OR UWS OR "vegetative state" OR VS OR "minimally conscious state" OR MCS OR "MCS+" OR "MCS-"). Since we were aiming for homogeneity regarding the clinical assessment used in the articles, we limited the results: the publication time was set to 2004 and later because since this time JFK Coma Recovery Scale was redefined as CRS-R. We used the Citation chaser tool (https://estech.shinyapps.io/citationchaser/) and performed backward chasing to identify other possible candidates for our article.

Our aim was to identify studies that include follow-up data of DOC patients which assess the prognostic value of EEG connectivity and network analyses, as well as the CRS-R scores.

### 2.2 Data collection and analysis

One author (SzD) performed the duplicate selection, after which two authors (SzD, AZ) independently performed title and abstract screening, including eligible articles. After the more vigorous full text assessment, they selected articles according to the predefined eligibility criteria. We excluded articles which (1) contained data about patient recovery in the acute phase (7–28 days, but we included those which mostly assessed prolonged DOC), (2) were case reports, or involved less than five patients, (3) used other clinical scales than CRS-R, since these scores underperform compared to CRS-R, (4) did not comprise follow-up data, (5) did not contain sensitivity/specificity/AUC results or these data could not be extracted. After unblinding the decision of the authors, any disagreement was resolved by discussion between the two authors. Automation tools were not used during the process.

Data extraction was performed on the final articles. We collected publication data, the type of prognostic measure (EEG connectivity measure/network analysis/spectral EEG results and CRS-R score), criterion of recovery, follow-up period, total number of patients, numbers of recovered and non-recovered patients, and prognostic accuracy of the examined measures (EEG and CRS-R). If raw data were available, prognostic accuracy was calculated. We contacted several authors for raw, patient-specific data. Given the small number of articles, there was no missing data in the identified categories.

If an article included different measure types for one outcome, we chose the one with the highest discriminative capacity.

Recovery was defined with different parameters by the authors, either as a two-point increase in the Glasgow Outcome Scale (GOS), or a category change between different states of DOC, namely UWS and MCS or MCS and conscious state (CS).

Methodological quality of the included studies was screened with QUAPAS tool [21], which is an adaptation of the QUADAS-2 tool for studies assessing prognostic accuracy. Risk of bias was determined in five distinct domains.

(1) participants: This domain addresses techniques for enrolling participants and preventing unwarranted exclusions when entering the study.
(2) index test: This domain encompasses potential sources of bias associated with defining, measuring, or interpreting the index test (in our case the CRS-R).
(3) outcome: This domain pertains to bias that could emerge from the definition, measurement method, or interpretation of the outcome.
(4) flow and timing: This domain concentrates on the inclusion of participants in the analysis, including the timing of the tests and considering the time horizon to capture the outcome.
(5) analysis: This domain focuses on the bias introduced by the analysis of the data itself, including handling missing data.

Each study was labelled with high, moderate, or low risk of bias.

### 2.3 Statistical analysis and data synthesis

The articles contained data on both outcome measures (EEG connectivity or network analysis and clinical scale). The effect measure was the AUC (Area Under the Roc Curve) of a given measure. The meta-analysis of the ROC curve provides a more comprehensive perspective on the predictive performance since it reveals the range of sensitivity and specificity pairs achievable as the threshold changes. Due to insufficient data, we were only able to conduct meta-analysis of the ROC curve for the CRS-R score. Statistical analyses were carried out using the R statistical software (version 4.1.2.). For all statistical analyses, a p-value of less than 0.05 was considered significant.

We collected the AUC values and their CI-s for each EEG measure. We estimated the standard errors of the AUC values from the confidence intervals. When a confidence interval was unavailable, we used the formula published in the article of Hanley et al. [22] In the case of the CRS-R measure, we calculated AUC and its standard error using raw data. The published CRS-R confidence interval in Stefan et al. (2018) differed substantially from the raw data-based standard error. Hence, instead of the published ones, we used the formula from the article of Hanley et al. [22] for the standard errors of the connectivity and power AUC values. The studies analysed two or three EEG measures on the same populations. To account for these correlations, we fitted a multivariable model using the rma.mv() function of the metafor R package. To circumvent the problem caused by the unknown correlations, first we took 0.6 the correlation between measures calculated on the same population and we supplemented the method with the robust approach of Pustejovsky et al. [23] implemented in the coef_test() function of the clubSandwich R package. Moreover, we repeated the approach under several between-study and within-study correlation assumptions. All sensitivity runs provided quite similar pooled AUC values and comparison p-values.

The population of Schorr et al. [24] overlaps with that of Stefan et al. [25]. For this reason, we omitted it from the main analysis. However, as another sensitivity analysis, we repeated the analysis mentioned above, including Schorr et al. [24] instead of Stefan et al. [25].

The availability of raw data in case of CRS-R made possible a more refined analysis. To get a better insight into the diagnostic performance, for several thresholds we calculated two-by-two contingency tables containing the true positive, false positive, false negative, and true negative values. To consider the trade-off between sensitivity and specificity when different threshold values are used, we created Summary Receiver Operating Characteristic (SROC) curves along with confidence intervals using the method introduced by Steinhauser et al. [26]. The advantage of this relatively new approach is that it handles the correlation between contingency tables from the same studies corresponding to different thresholds.

For thresholds 6 and 7, we also directly pooled sensitivity and specificity using the univariate generalized mixed-effect approach of Stijnen et al. [27].

We also compared the mean age of the recovered and non-recovered patients using classical univariate inverse-variance random-effects meta-analysis with restricted maximum likelihood estimation.

Due to the small number of available studies, publication bias could not be assessed.

## 3 Results

### 3.1 Search and selection

#### 3.1.1 Prisma flowchart

**Fig. 2:**
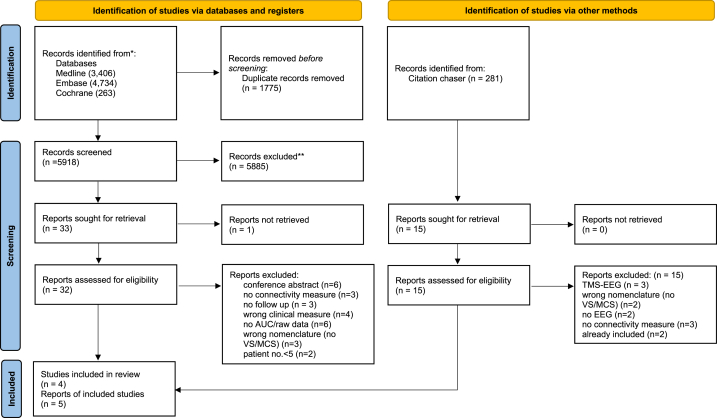
PRISMA flowchart of the selection of article.

Our systematic search resulted in 32 full text articles. From those, we excluded articles for three main reasons: the use of a clinical scale other than CRS-R, no diagnostic accuracy testing, or low sample size. Ultimately, five articles met all our inclusion criteria, containing both CRS-R scores and EEG connectivity or graph theory measures, enabling us to assess the difference in prognostic power of the studied measures in the same population. Detailed information about the article selection process is included in the PRISMA flowchart (Fig. 2).

#### 3.1.2 Basic characteristics of included studies

The 5 articles included 280 patients. Two articles [24,25] had overlapping populations, which we discovered after the raw data analysis. For this reason, we included Stefan et al. [25] only in the primary analysis, since it assessed more EEG markers (power and connectivity), but we conducted an additional analysis using the data from Schorr et al. [24] (Supp. Fig. 3) It should be noted that previous meta-analyses have ignored this statistically important factor [20]. Following the exclusion of this patient cohort, our study sample size was reduced to 222 individuals. Of this group, a total of 79 patients had positive outcomes, according to the different recovery criteria of the articles. Follow-up time in the articles ranged from 42 days to over 3 years. Since the articles assessed EEG spectral power and automated outcome prediction methods besides CRS-R, connectivity, and graph-theory measures, we decided to include them in the analysis. The articles contained either AUC values, sensitivity, specificity values of the prognostic capacity or raw, patient specific data of the studied variables. We received patient specific connectivity (Quadratic Phase Self-Coupling [QPSC]) data from one of the authors (Yang Bai), to calculate AUC values.(see. Table 1)

### 3.2 Risk of bias assessment

**Fig. 3:**
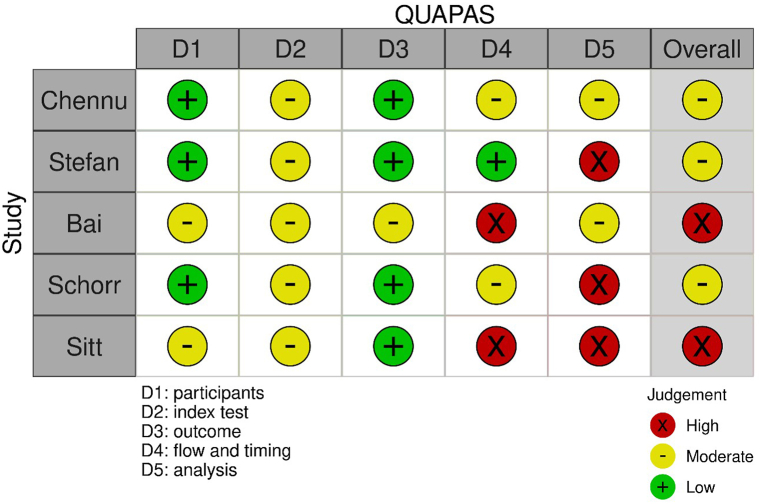
Risk of Bias assessment - QUAPAS tool: Quality assessment of the articles proved moderate to high overall bias of the reviewed articles.

Risk of bias assessment was performed with the QUAPAS tool. It indicated a moderate to high overall risk of bias. Bias was primarily attributed to the ‘flow and timing’ and the ‘analysis’ domains, since in most of the studies the follow-up time was not sufficient for capturing outcome, and not all enrolled participants were included in the final analysis (Fig. 3).

### 3.3 Prognostic capacity of the different measures

#### 3.3.1 EEG connectivity and network analysis measures

**Fig. 4:**
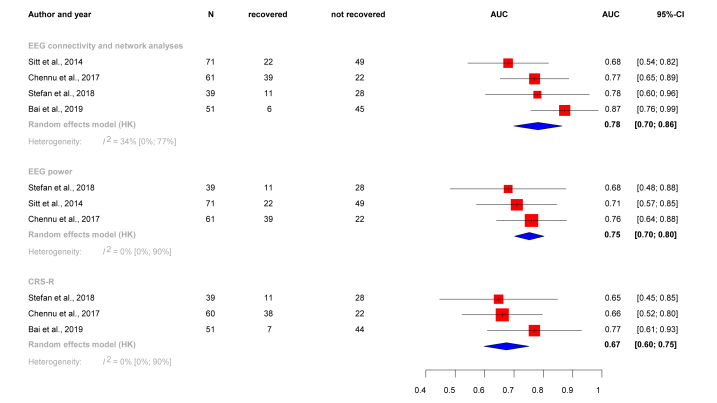
Forest-plot displaying prediction of outcome. The effect measure was the discriminative capacity (AUC) of EEG connectivity measures and network metrics, EEG spectral power, and clinical scale. The red squares show the estimates of the effects in each study; horizontal lines are the CI-s, while blue diamonds show the estimates of the overall effects. EEG connectivity measures and network metrics perform significantly better (AUC = 0.78, CI: 0.70–0.86) than CRS-R (0.67, CI:0.60–0.75) (p = 0.00708). (For interpretation of the references to colour in this figure legend, the reader is referred to the Web version of this article.)

The estimated discriminative capacity of EEG connectivity and network analysis markers in predicting prognosis was 78 % (AUC 0.78, CI: 0.70–0.86). Within the markers, we included coherence, modularity, and Phase Lag Index (PLI) (Fig. 4). Difference between the discriminative capacity of connectivity measures and network metrics subgroup and CRS-R subgroup was significant (p = 0.00708), and non-significant from the power subgroup (p = 0.39431). As the article of Stefan et al. [25] has demonstrated high performance of a network metric (clustering coefficient of alpha band and beta band) besides connectivity measure (coherence), we conducted an additional quantitative analysis. To mitigate further potential biases, we have repeated the quantitative analysis utilizing the dataset of Schorr et al. [24]. Assessing different types of measures or datasets did not significantly change the pooled discriminative capacity, nor their relationship to each other (Supp. material, Fig. 2, Fig. 3).

#### 3.3.2 Prognostic capacity of spectral power

As for the EEG spectral power, this measure was slightly less discriminative than connectivity measures and network metrics (AUC 0.75, CI:0.70–0.80). The difference between the two subgroups was non-significant (p = 0.39431). Spectral power did not perform significantly better than CRS-S scale (p = 0.08189) (Fig. 4).

#### 3.3.3 Prognostic capacity of CRS-R scale

The evaluation of the CRS-R varied across the reviewed articles, with some articles lacking mention of the repetition of assessments, which is a critical aspect of the assessment protocol (See Results, Table 2.).

**Table 1:** Basic characteristics of the included articles (MCS: minimally conscious state, UWS: unresponsive wakefulness syndrome, DOC definition: how DOC was diagnosed, several articles used other clinical scales, or clinical consensus, recovered/not recovered: criterion of recovery was different in the articles, positive outcome meant GOS >2 or recovery from UWS to MCS or MCS to consciousness).

Number	Publication data		Measure type	EEG density	Age (years)	Follow-up period	DOC definition	All patient number	MCS	UWS	recovered	not recovered
	First Author	Year of publication										
1	Chennu	2017	delta modularity (non-traumatic)	256 channels	38,6 ± 15,3	1 year	CRS-R	61	NA	NA	39	22
			delta clustering coefficient (traumatic)									
			relative theta power									
2	Stefan	2018	ApEn in alfa band	256 channels	51,85 ± 17,57	589,26 ± 1125,32 days	CRS-R	39	0	39	11	28
			coherence theta band									
			complex network analysis, clustering coefficients, beta band									
			complex network analysis, clusteing coefficient alfa band									
			alfa power									
			delta power									
3	Bai	2019	Frontal QPSC-theta, Quadratic Phase Self Coupling	62 channel	MCS: 45,35 UWS: 45.68	3 month	CRS-R	51	20	31	7	44
4	Schorr	2016	parietal coherence in the theta band	256 channels	MCS: 50,3 ± 10 UWS: 50,2 ± 17,1	12 month	CRS-R	58	0	58	13	45
			parietal coherence in the delta band									
			fronto-parietal coherence in the alpha band									
			fronto-parietal coherence in the theta band									
5	Sitt	2014	delta PLI	256 channel	48,3 ± 17	max. 42 days	CRS-R	71	0	71	22	49
			SE									
			theta power

**Table 2:** Risk of bias assessment of articles based on the protocol used to diagnose patients with CRS-R. Three out of five articles have high risk of bias.

Article	Assessment protocol	Bias
Chennuet al.	Diagnosis was based on the highest score obtained over five to seven CRS-R assessments during the day.	low
Schorr et al.	State of consciousness was assessed independently by two experienced raters with the CRS-R.	moderate
Stefan et al.	State of consciousness both at baseline and follow-up was assessed with the CRS-R.	high
Bai et el.	Clinical assessment was carefully conducted by trained neurologists using the CRS-R.	high
Sitt et al.	Clinical evaluation of consciousness was based on the French version of the Coma Recovery Scale Revised (CRS-R) scale, and careful neurological examination by trained neurologists.	high

The CRS-R's ability to predict outcome was moderate. (AUC: 0.67, CI: 0.60–0.75).

**Fig. 5:**
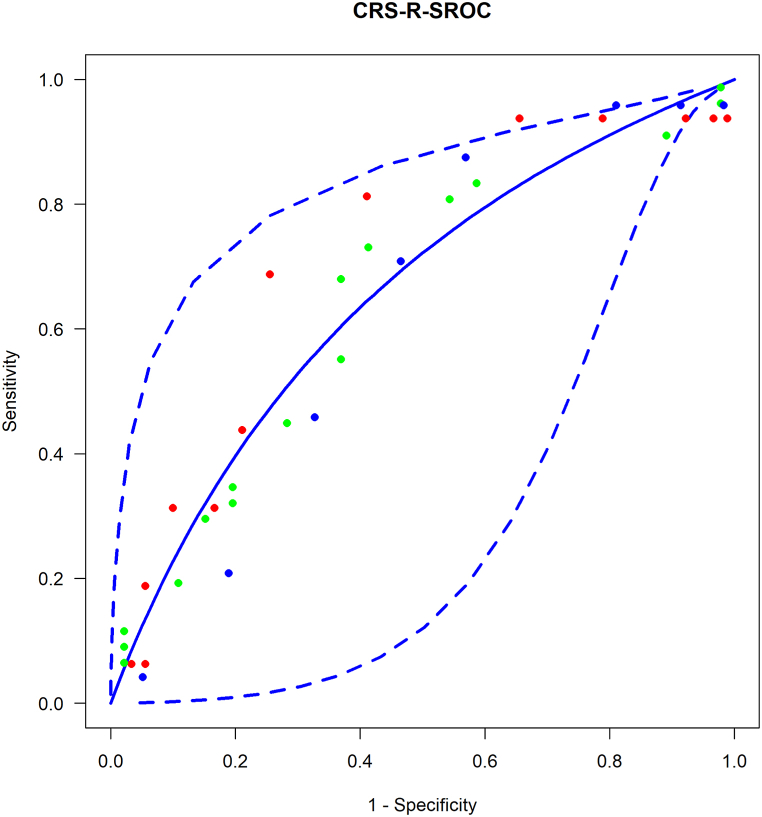
Roc curve with 95 % confidence interval, showing the discriminative capacity of the CRS-R score. The different coloured dots represent different articles, with each dot representing an estimated sensitivity and specificity value at a given cut-off value.

**Fig. 6:**
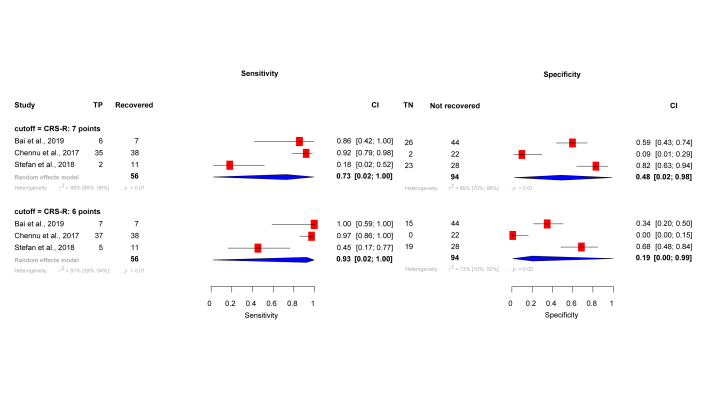
Forest-plot of sensitivity and specificity analyses showing the discriminative capacity of CRS-R scale. We assessed the sensitivity and specificity at different thresholds. High sensitivity is associated with moderate to low specificity, this can be attributed to the limited discriminative capacity of this measure.

We tested the CRS-R parameter's sensitivity and specificity at different cut-off values (Fig. 5). Meta-ROC curve shows the trade-off between sensitivity and specificity as the employed threshold changes. High sensitivity (93 % (CI: 0,2–100 %)) with very low specificity (19 % (CI: 0–99 %)) could be reached at a cut-off value of CRS-R score 6; however, relatively high sensitivity (73 % (CI: 0,2–100 %)), with adequate specificity (48 % (CI: 0,2–98 %)) was obtained at a cut-off value of CRS-R score 7 (Fig. 6).

#### 3.3.4 Prognostic capacity of age

We assessed patients’ age in the recovered and non-recovered groups. According to our analysis mean difference of age was not a significant factor influencing the outcome, however, it should be noted that one particular article (Bai et al. [28]) reported results that were contrary to the trend observed in the other two studies (Supp. material, Fig. 1).

#### 3.3.5 Automated outcome prediction tools

Chennu et al. [29] created a support vector machine (SVM) consisting of two features: delta modularity and clustering coefficients. They reached 92 % sensitivity and 64 % specificity in discriminating positive and negative outcome of patients, defined by their GOS-E scores. Automated outcome prediction in the work of Stefan et al. [25] used three different attributes: frequency of microstate A in the 2–20 Hz frequency band, path length obtained from thresholding alpha coherence, and clustering coefficient obtained from thresholding alpha coherence. The discriminating capacity of the pooled measures reached 92 % (AUC = 92 % ± 4 %). The prognostic capacity of these machine learning algorithms was higher than the ones observed in the group of connectivity and graph-theory measures.

### 3.4 Publication bias and heterogeneity

Overall heterogeneity of the subgroups was low, although CI was large for all measures. The statistical reason for this high uncertainty is the small sample size. The heterogeneity observed in the calculation of diagnostic accuracy for the CRS-R is considerable, attributable to the dissimilarities in the populations examined in the different studies. Schorr et al. [24] only included UWS patients, while the other two articles included MCS and UWS patients as well.

## 4 Discussion

Our systematic review shows that EEG connectivity and network metrics perform better in prognostication of outcome in patients with prolonged DOC compared to clinical scales, but the best results can be achieved with automated prediction tools by using a combination of various metrics.

In our study, age was not a good outcome predictor, although most earlier studies showed a strong correlation between younger age and recovery [30,31]. In one study [28], older age was positively associated with good recovery; however, in the group of recovered patients, brain haemorrhage or traumatic brain injury was the most frequent etiology, while the non-recovered group included several patients with anoxia. This contrast to previous research regarding age as a prognostic factor may be attributed to the small sample size, and the relatively short follow-up period.

Although CRS-R can reliably diagnose UWS and MCS [29], its overall predictive capacity remains moderate, however our results suggest that its best accuracy can be achieved at CRS-R total score ≥6, which is in line with previous results [32]. Taking into consideration that the CRS-R scale is broadly available its high sensitivity should be valued. There is no specific threshold for what is considered a favourable AUC score. Prognostic capacity of CRS-R in our scenario is insufficient to warrant discontinuation of treatment for patients with disorders of consciousness (DOC); however, it can provide valuable additional information and facilitate communication with the patients' family.

The total CRS-R scores are subject to bias due to limited information about the assessment surroundings in the reviewed articles. Only one article provided comprehensive details about the assessment protocol where the diagnosis was based on the highest values obtained from 5 to 7 assessments [29] (Table 2.).

The predictive ability of EEG spectral power measures is satisfactory, and not significantly lower than that of connectivity and graph-theory measures. Stefan et al. [25] reported that higher alpha power indicates recovery, while Sitt et al. [33] and Chennu et al. [29] found that higher theta power may indicate good prognosis. EEG spectral studies show that in DOC patients, high alpha and low delta power are negatively correlated with the severity of the disease and can predict good recovery [[34], [35], [36], [37]]. Alpha power is positively correlated, while delta power is negatively correlated with CRS-R scores [38]. In DOC, higher power in the theta frequency band (5–8 Hz) indicates survival [39].

EEG connectivity measures accurately prognosticate and describe higher order cognitive functions such as consciousness or cognitive function in neurodegenerative diseases [14,15], due to the fact that complex brain functions depend on a fine balance between local and global integration and not only on the behaviour of individual machineries. Coherence effectively predicted the outcome even though power analysis did not show a significant difference between the recovered and non-recovered patients [24]. Nevertheless, data suggest that power spectral density and selected network metrics are correlated and should not be published separately [40]. To verify the superiority of connectivity and network metrics, more studies directly comparing the two methods are needed (in accordance with the Cochrane Handbook - chapter 15 [41]).

Connectivity measures and network metrics found during our systematic search can be divided into two groups based on the frequency band on which the functional interactions were observed. Sitt et al. [33], and Chennu et al. [29] assessed connectivity in the delta band and the delta network while Stefan et al. [25], Schorr et al. [24], and Bai et al. [28] analysed the theta band and the theta network.

Chennu et al. [29] published outcomes separately on traumatic and non-traumatic aetiologies. They found that higher modularity in the delta network of non-traumatic patients (AUC 0.77) and higher microscale clustering coefficients in the delta network of traumatic patients (AUC 0.78) were predictors of positive outcome.

Sitt et al. [33] published data after a short follow-up of patients and found that smaller PLI in the delta frequency band correlated with better outcome.

Regarding the delta network, it seems that the lack of large-scale synchronous delta oscillations and higher network modularity indicates good recovery chances [29]. Regarding network organization, Kustermann et al. [42] found similar results in the outcome of acute coma. According to their results, higher modularity favours recovery, suggesting that mesoscale segregation precedes regaining consciousness.

Differences in the theta network were described with coherence and QPSC and higher coherence in the theta band (Stefan et al. [25], Schorr et al. [24]), while lower frontal theta bicoherence (Bai et al. [28]) was found which might suggest successful memory encoding. Theta synchronization occurs during enhanced cognition, successful memory encoding [43]. The articles did not apply age adjustment, which could have a significant impact, given the well-established age-related alterations in connectivity and structural network changes [44]. It is also identified that theta activity decline and theta connectivity changes in the midline-frontal areas may indicate aging [45].

Clustering coefficient calculated from the alpha and beta band demonstrated strong performance, higher clustering coefficient meaning better chance for recovery (Stefan et al. [25]). This difference was seen in the theta band as well, but less prominently, while clustering coefficient calculated from the delta band could not predict outcome. Recent data showed in a 2-year long follow-up, that clustering coefficient calculated from the alpha band is the strongest predictor of clinical outcome of the 42 high density EEG measures assessed [46].

## 5 Strengths and limitations

This is the first meta-analysis conducted on the prognostic accuracy of EEG connectivity markers and network metrics in patients with DOC. It employs strict methodology, including a systematic search strategy and strict inclusion criteria. With a relatively large sample size, the study provides reliable and generalizable conclusions, demonstrating the efficacy of the studied EEG markers in DOC patients. The findings have important clinical implications, such as informing clinical practice, to integrate EEG markers into guidelines and protocols for the assessment and management of DOC patients. The findings highlight research priorities and encourage further investigation.

The heterogeneity of the studied variables is high, but since we compared the prognostic capacity of clinical scales and computational EEG methods, we believe that pooling these subcategories does not distort the results.

The main limitation of our meta-analysis is the few number of studies included causing a statistically small sample size, which is due to: 1) the low prevalence of the studied disorders of consciousness (UWS and MCS); 2) the difficulty of follow-up in this patient population; and 3) the complexity of the EEG connectivity methods used by the authors. Notwithstanding these drawbacks, our sample size may still be considered clinically relevant. There is a notable publication bias for the statistically significant and highly predictive methods, which makes the comparison difficult.

Heterogeneity is also high due to the different lengths of follow-up periods (from 42 days to 3 years). Conducting subgroup analyses to further elucidate these differences was not possible due to the lack of data.

All studies used high density EEG, which can cause clinical heterogeneity between the articles and can limit the clinical use of the found measures. It was proposed beforehand that network metrics accurately describe functional network changes in DOC, but with low density EEG they might not be able to correctly capture all features of brain connectivity [47]. However, to date, there is a no available data regarding potential disparities in predictive abilities of connectivity or network metric results when comparing 32 and 64 channel configurations.

## 6 Future perspective

### 6.1 The importance of repeated measures

Repetition in individual patients

Bedside EEG can easily be repeated to uncover changes in arousal and in the motivational state of the patient, which is of paramount importance in this patient population.

Replication across studies

EEG connectivity studies in DOC are in the exploratory phase. Publication bias should be avoided, and non-significant or contradictory results should be published, since expanding knowledge about connectivity requires awareness that the results can be replicated. Indeed, as Mike X Cohen states, (Analyzing time series data, page 546, chapter 38.) “replication of (EEG) studies is important because experiments are imperfect.”. Knowing the underlying reasons for incongruity, is important, since these might indicate the existence of different subgroups of patients [48]. A recent review shows high heterogeneity of EEG-based quantitative techniques in prognostic studies in DOC, and the lack of consensus in experimental settings [49]. According to their findings the heterogeneity can arise from the differences in data pre-processing, domain analysis or feature extraction.

Data pre-processing is one of the most prominent and heterogeneous part of the analysis, because of the different number of electrodes used, filtering, artefact rejection techniques and epoch selection. Between our included studies all were high density EEG recordings, which makes the translation of the results hard. Filtering among our articles was done by low and high pass filters and notch filter. Artefact rejection was be done visually or automatically or semi-automatically by Independent Component Analysis (ICA). Both filtering and artefact rejection introduce the potential risk of inadvertently eliminating biological signals. Epoch length and selection can differ too, which can bias the connectivity estimates [50]. For the optimization and standardization of EEG connectivity measures important propositions have been put forward [51]. The variances in EEG analysis characteristics among the included articles are documented in Supplementary Table 1.

Replicating studies using 32-channel EEG configurations is essential to facilitate the translation of the identified metrics into clinical practice. While univariate markers can reach high accuracy in prognostication, the best predictive results can be achieved by combining different EEG analysis methods. Combining methods with machine-learning algorithm or support vector machines has higher discriminative capacity than univariate markers [14]. It was shown that the accuracy of different EEG markers changed according to sensor number, position and epoch length; however, the classifier itself was immune to these changes [14].

Sitt et al. [33] suggested choosing the best-performing marker from the same patient population and using cross-validation techniques for the verification of the marker to avoid the overfitting. For this purpose, Chennu et al. [29] used fourfold cross-validation, while Stefan et al. [25] used tenfold cross-validation. The validation analyses not only suggest a successful implementation of the EEG-measures reported [33] but also extend our understanding of their practical properties such as their dependency on acquisitions and setups.

Choosing the best-performing markers (most relevant attributes) is an important issue, since it can improve prognostication. Attribute selection is a well-known problem in the field of machine learning, and various solutions are available, including forward selection, backward elimination, or quick reduction algorithms. Meanwhile, user friendly interfaces can assist the application of machine learning algorithm to clinical usage. An example of this is WEKA software, which has already been used for outcome prediction in neurological diseases [52].

Engemann et al. go a step further and advocate for the application of neuroscience discoveries and data science tools to clinical practice, by creating an automatic prediction system for clinicians capable of analysing raw EEG data of DOC patients [14].

Appropriate risk estimation is also essential for informed clinical decision-making. A natural requirement for any risk-estimating model is that predictions are well-calibrated, see Van Calster et al. [53]. None of the studies provided calibrated risk estimation models; nevertheless, it is an important direction in the case of complex models.

## 7 Conclusion

EEG connectivity measures and network metrics can be used with good accuracy in outcome prognostication in patients with DOC. Multivariate methods comprising EEG connectivity markers promise better predictive ability, but the potential pitfalls, such as overfitting, should be considered. The CRS-R demonstrates a moderate prognostic capacity, but considering its widespread availability, it warrants acknowledgment as a valuable tool.

## Data availability

Data is available upon request from the corresponding author, DSZ.

## CRediT authorship contribution statement

**Danuta Szirmai:** Writing – review & editing, Writing – original draft, Investigation, Formal analysis, Data curation, Conceptualization. **Arashk Zabihi:** Formal analysis, Data curation. **Tamás Kói:** Writing – review & editing, Writing – original draft, Supervision, Software, Formal analysis. **Péter Hegyi:** Writing – review & editing, Supervision, Methodology, Conceptualization. **Alexander Schulze Wenning:** Writing – review & editing, Supervision, Methodology. **Marie Anne Engh:** Methodology. **Zsolt Molnár:** Writing – review & editing, Supervision. **Gábor Csukly:** Writing – review & editing, Supervision, Data curation, Conceptualization. **András Attila Horváth:** Writing – review & editing, Supervision, Methodology, Investigation, Conceptualization.

## Declaration of competing interest

The authors declare that they have no known competing financial interests or personal relationships that could have appeared to influence the work reported in this paper.

## Appendix A Supplementary data

Supplementary materials Fig. 1 Forest plot depicting the meta-analysis of age mean difference in the outcome, showing non-significant results. Heterogeneity is high because in one article (Bai et al. [28]) the mean age was higher in the recovered group. Supplementary materials Fig. 1

Supplementary materials Fig. 2 Forest-plot showing the prediction of outcome. The effect measure was the discriminative capacity (AUC) of EEG connectivity measures and network metrics, EEG spectral power and clinical scale. The red square stands for the estimates of the effect in each study, horizontal lines are the CI-s, while the blue diamond shows the estimate of the overall effect. This analysis was conducted using another measure from the Stefan et al. [25] study (clustering coefficients, calculated from alpha coherence). Supplementary materials Fig. 2

Supplementary materials Fig. 3 Forest-plot showing the prediction of outcome. The effect measure was the discriminative capacity (AUC) of EEG connectivity measures and network metrics, EEG spectral power and clinical scale. The red square stands for the estimates of the effect in each study, horizontal lines are the CI-s, while the blue diamond shows the estimate of the overall effect. This analysis was conducted using the data from Schorr et al. [24]. Supplementary materials Fig. 3

Supplementary materials Table 1 EEG analysis characteristics of the included articles (ICA: Independent Component Analysis, NA: Not Applicable, not mentioned in the article). Supplementary materials Table 1

## References

[1] Caroline Schnakers S.L. (2018).

[2] Giacino J.T., Ashwal S., Childs N., Cranford R., Jennett B., Katz D.I. (2002). The minimally conscious state: definition and diagnostic criteria. Neurology.

[3] Stepan C., Haidinger G., Binder H. (2004). Prevalence of persistent vegetative state/apallic syndrome in Vienna. Eur. J. Neurol..

[4] van Erp W., Lavrijsen J.C.M., Van De Laar F.A., Vos P.E., Laureys S., Koopmans R.T. (2014). The vegetative state/unresponsive wakefulness syndrome: a systematic review of prevalence studies. Eur. J. Neurol..

[5] Estraneo A., Moretta P., Fau - Loreto V., Loreto V., Fau - Lanzillo B., Lanzillo B., Fau - Santoro L., Santoro L., Fau - Trojano L., Trojano L. (2010). Late recovery after traumatic, anoxic, or hemorrhagic long-lasting vegetative state.

[6] Turner-Stokes L., Thakur V., Dungca C., Clare C., Alfonso E. (2022). End-of-life care for patients with prolonged disorders of consciousness following withdrawal of life-sustaining treatment: Experience and lessons from an 8-year cohort.

[7] Estraneo A., Fiorenza S., Magliacano A., Formisano R., Mattia D., Grippo A. (2020). Multicenter prospective study on predictors of short-term outcome in disorders of consciousness. Neurology.

[8] Portaccio E., Morrocchesi A., Romoli A.M., Hakiki B., Taglioli M.P., Lippi E. (2018). Score on Coma Recovery Scale-Revised at admission predicts outcome at discharge in intensive rehabilitation after severe brain injury. Brain Inj..

[9] Kalmar K., Giacino J.T. (2005). The JFK Coma Recovery Scale-Revised.

[10] Lucca L.A.-O., Lofaro D., Pignolo L., Leto E., Ursino M., Cortese M.D. (2019). Outcome prediction in disorders of consciousness: the role of coma recovery scale revised.

[11] Schnakers C., Giacino J., Kalmar K., Piret S., Lopez E., Boly M., Malone R., Laureys S. (2006). Does the FOUR score correctly diagnose the vegetative and minimally conscious states?.

[12] Schnakers C., Majerus S., Fau - Giacino J., Giacino J., Fau - Vanhaudenhuyse A., Vanhaudenhuyse A., Fau - Bruno M.-A., Bruno Ma Fau - Boly M., Boly M., Fau - Moonen G. (2008). A French validation study of the Coma Recovery Scale-Revised (CRS-R).

[13] Bender A., Jox R.J., Grill E., Straube A., Lulé D. (2015). Persistent vegetative state and minimally conscious state: a systematic review and meta-analysis of diagnostic procedures. Dtsch Arztebl Int.

[14] Denis Engemann FR., King Jean-Remi, Mainak Jas, Alexandre Gramfort S.D., Naccache Lionel, Sitt Jacobo D. (2015). Machine Learning and Neuroscience (Stamlins 2015).

[15] Wang R., Wang J., Yu H., Wei X., Yang C., Deng B. (2015). Power spectral density and coherence analysis of Alzheimer's EEG. Cogn Neurodyn.

[16] Horvath A., Szucs A., Csukly G., Sakovics A., Stefanics G., Kamondi A. (2018). EEG and ERP biomarkers of Alzheimer's disease: a critical review.

[17] Stam C.J. (2014). Modern network science of neurological disorders.

[18] Kondziella D.A.-O., Bender A.A.-O., Diserens K., van Erp W.A.-O., Estraneo A.A.-O., Formisano R.A.-O. (2020). European Academy of Neurology guideline on the diagnosis of coma and other disorders of consciousness..

[19] Giacino J.T., Katz D.I., Schiff N.D., Whyte J., Ashman E.J., Ashwal S. (2018). Practice guideline update recommendations summary: Disorders of consciousness: Report of the Guideline Development, Dissemination, and Implementation Subcommittee of the American Academy of Neurology; the American Congress of Rehabilitation Medicine; and the National Institute on Disability, Independent Living, and Rehabilitation Research.

[20] Song M., Yang Y., Yang Z., Cui Y., Yu S., He J. (2019). Prognostic models for prolonged disorders of consciousness: an integrative review.

[21] Lee J.A.-O., Mulder F., Leeflang M.A.-O., Wolff R.A.-O., Whiting P.A.-O., Bossuyt P.A.-O. (2022). QUAPAS: An Adaptation of the QUADAS-2 Tool to Assess Prognostic Accuracy Studies.

[22] Hanley J.A., McNeil B.J. (1982). The meaning and use of the area under a receiver operating characteristic (ROC) curve. Radiology.

[23] Pustejovsky J.E., Tipton E. (2022). Meta-analysis with robust variance estimation: expanding the range of working models. Prev. Sci..

[24] Schorr B., Schlee W., Arndt M., Bender A. (2016). Coherence in resting-state EEG as a predictor for the recovery from unresponsive wakefulness syndrome. J. Neurol..

[25] Stefan S., Schorr B., Lopez-Rolon A., Kolassa I.T., Shock J.P., Rosenfelder M. (2018). Consciousness indexing and outcome prediction with resting-state EEG in severe disorders of consciousness. Brain Topogr..

[26] Steinhauser S., Schumacher M., Rücker G. (2016). Modelling multiple thresholds in meta-analysis of diagnostic test accuracy studies. BMC Med. Res. Methodol..

[27] Stijnen T., Hamza T.H., Özdemir P. (2010). Random effects meta-analysis of event outcome in the framework of the generalized linear mixed model with applications in sparse data. Stat. Med..

[28] Bai Y., Xia X., Wang Y., He J., Li X. (2019). Electroencephalography quadratic phase self-coupling correlates with consciousness states and restoration in patients with disorders of consciousness. Clin. Neurophysiol..

[29] Chennu S., Annen J., Wannez S., Thibaut A., Chatelle C., Cassol H. (2017). Brain networks predict metabolism, diagnosis and prognosis at the bedside in disorders of consciousness.

[30] Arnaldi D., Terzaghi M., Cremascoli R., De Carli F., Maggioni G., Pistarini C. (2016). The prognostic value of sleep patterns in disorders of consciousness in the sub-acute phase. Clin. Neurophysiol..

[31] Song M., Yang Y., He J., Yang Z., Yu S., Xie Q. (2018). Prognostication of chronic disorders of consciousness using brain functional networks and clinical characteristics. Elife.

[32] Estraneo A., Moretta P., Fau - Loreto V., Loreto V., Fau - Lanzillo B., Lanzillo B., Fau - Cozzolino A., Cozzolino A., Fau - Saltalamacchia A., Saltalamacchia A., Fau - Lullo F. (2013). Predictors of recovery of responsiveness in prolonged anoxic vegetative state.

[33] Sitt J.D., King J.R., El Karoui I., Rohaut B., Faugeras F., Gramfort A. (2014). Large scale screening of neural signatures of consciousness in patients in a vegetative or minimally conscious state. Brain.

[34] Kotchoubey B., Pavlov Y.G. (2018). A Systematic Review and Meta-Analysis of the Relationship Between Brain Data and the Outcome in Disorders of Consciousness.

[35] Leon-Carrion J., Martin-Rodriguez J.F., Damas-Lopez J., Barroso y Martin J.M., Dominguez-Morales M.R. (2009). Delta–alpha ratio correlates with level of recovery after neurorehabilitation in patients with acquired brain injury. Clin. Neurophysiol..

[36] Babiloni C., Sarà M., Vecchio F., Pistoia F., Sebastiano F., Onorati P. (2009). Cortical sources of resting-state alpha rhythms are abnormal in persistent vegetative state patients. Clin. Neurophysiol..

[37] Rossi Sebastiano D., Panzica F., Visani E., Rotondi F., Scaioli V., Leonardi M. (2015). Significance of multiple neurophysiological measures in patients with chronic disorders of consciousness.

[38] Chennu S., Finoia P., Kamau E., Allanson J., Williams G.B., Monti M.M. (2014). Spectral signatures of reorganised brain networks in disorders of consciousness.

[39] Fingelkurts A.A., Fingelkurts A.A., Bagnato S., Boccagni C., Galardi G. (2011). Life or death: prognostic value of a resting EEG with regards to survival in patients in vegetative and minimally conscious States.

[40] Demuru M., La Cava S.M., Pani S.M., Fraschini M. (2020). A comparison between power spectral density and network metrics: an EEG study. Biomed. Signal Process Control.

[41] Cochrane Handbook for Systematic Reviews of Interventions version 6.3 (updated February 2022). www.training.cochrane.org/handbook2022.

[42] Kustermann T., Ata Nguepnjo Nguissi N., Pfeiffer C., Haenggi M., Kurmann R., Zubler F. (2020). Brain functional connectivity during the first day of coma reflects long-term outcome. Neuroimage: Clinical..

[43] Solomon E.A., Kragel J.E., Sperling M.R., Sharan A., Worrell G., Kucewicz M. (2017). Widespread theta synchrony and high-frequency desynchronization underlies enhanced cognition. Nat. Commun..

[44] Meunier D., Achard S., Morcom A., Bullmore E. (2009). Age-related changes in modular organization of human brain functional networks.

[45] Tóth B., Kardos Z., File B., Boha R., Stam C.J., Molnár M. (2014). Frontal midline theta connectivity is related to efficiency of WM maintenance and is affected by aging.

[46] Bareham C.A., Roberts N., Allanson J., Hutchinson P.J.A., Pickard J.D., Menon D.K. (2020). Bedside EEG predicts longitudinal behavioural changes in disorders of consciousness. Neuroimage: Clinical..

[47] O'Donnell A., Pauli R., Banellis L., Sokoliuk R., Hayton T., Sturman S. (2021). The prognostic value of resting-state EEG in acute post-traumatic unresponsive states. Brain Commun.

[48] Sinitsyn D.O., Poydasheva A.G., Bakulin I.S., Legostaeva L.A., Iazeva E.G., Sergeev D.V. (2021). Machine Learning in the Diagnosis of Disorders of Consciousness: Opportunities and Challenges.

[49] Ballanti S., Campagnini S., Liuzzi P., Hakiki B., Scarpino M., Macchi C. (2022). EEG-based methods for recovery prognosis of patients with disorders of consciousness: a systematic review. Clin. Neurophysiol..

[50] Bastos A.M., Schoffelen J.M. (2015). A tutorial review of functional connectivity analysis methods and their interpretational pitfalls. Front. Syst. Neurosci..

[51] Miljevic A., Bailey N.W., Vila-Rodriguez F., Herring S.E., Fitzgerald P.B. (2021).

[52] Wang H.L., Hsu W.Y., Lee M.H., Weng H.H., Chang S.W., Yang J.T. (2019). Automatic Machine-Learning-Based Outcome Prediction in Patients With Primary Intracerebral Hemorrhage.

[53] Van Calster B., McLernon D.J., van Smeden M., Wynants L., Steyerberg E.W., Bossuyt P. (2019). Calibration: the Achilles heel of predictive analytics. BMC Med..

