# Supplementary material for: EEG connectivity and network analyses predict outcome in patients with disorders of consciousness – A systematic review and meta-analysis
Source: Heliyon. 2024 May 15;10(10):e31277. doi: 10.1016/j.heliyon.2024.e31277 (PMC11141356; doi:10.1016/j.heliyon.2024.e31277)
Supplement: Supplementary materials Fig. 1 — Forest plot depicting the meta-analysis of age mean difference in the outcome, showing non-significant results. Heterogeneity is high because in one article (Bai et al. [28]) the mean age was higher in the recovered group. [file mmc1.pptx]

## Slide 1
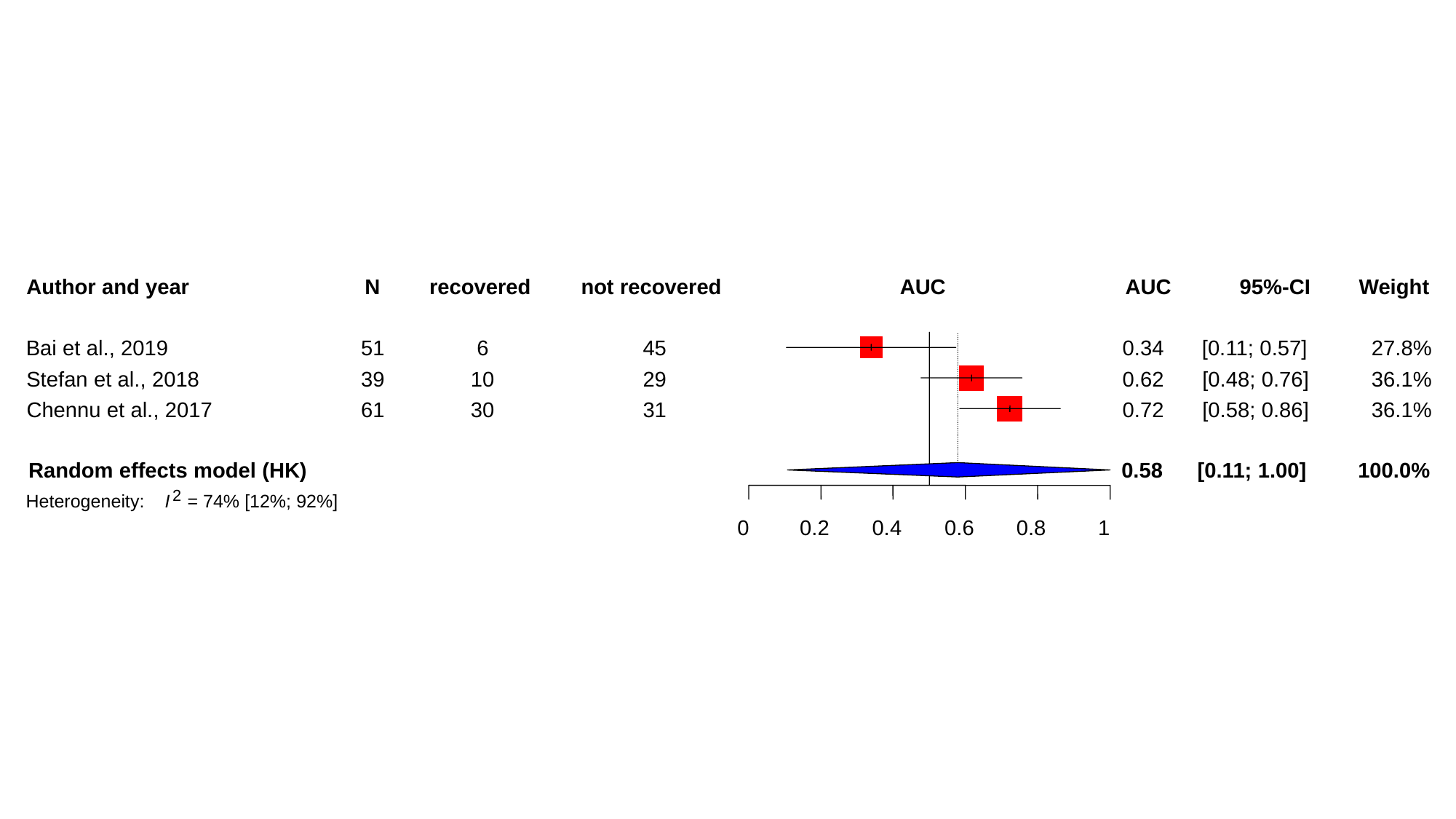

Author and year
N
recovered
not recovered
AUC
AUC
95%-CI
Weight
Bai et al., 2019
51
6
45
0.34
[0.11; 0.57]
27.8%
Stefan et al., 2018
39
10
29
0.62
[0.48; 0.76]
36.1%
Chennu et al., 2017
61
30
31
0.72
[0.58; 0.86]
36.1%
Random effects model (HK)
0.58
[0.11; 1.00]
100.0%
2
Heterogeneity:
I
 = 74% [12%; 92%]
0
0.2
0.4
0.6
0.8
1
