# Supplementary material for: EEG connectivity and network analyses predict outcome in patients with disorders of consciousness – A systematic review and meta-analysis
Source: Heliyon. 2024 May 15;10(10):e31277. doi: 10.1016/j.heliyon.2024.e31277 (PMC11141356; doi:10.1016/j.heliyon.2024.e31277)
Supplement: Supplementary materials Fig. 2 — Forest-plot showing the prediction of outcome. The effect measure was the discriminative capacity (AUC) of EEG connectivity measures and network metrics, EEG spectral power and clinical scale. The red square stands for the estimates of the effect in each study, horizontal lines are the CI-s, while the blue diamond shows the estimate of the overall effect. This analysis was conducted using another measure from the Stefan et al. [25] study (clustering coefficients, calculated from alpha coherence). [file mmc2.pptx]

## Slide 1
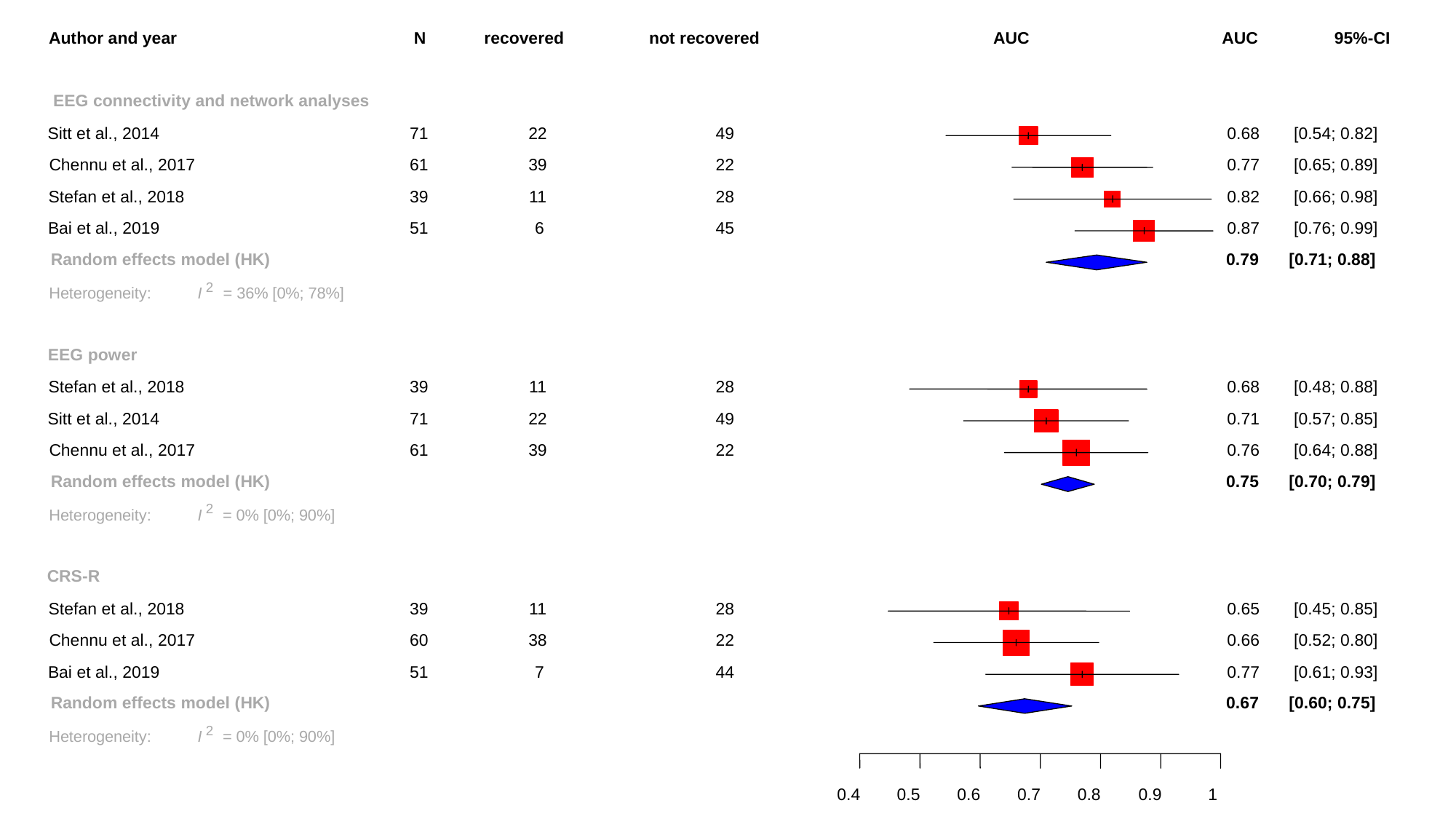

Author and year
N
recovered
not recovered
AUC
AUC
95%-CI
EEG connectivity and network analyses
Sitt et al., 2014
71
22
49
0.68
[0.54; 0.82]
Chennu et al., 2017
61
39
22
0.77
[0.65; 0.89]
Stefan et al., 2018
39
11
28
0.82
[0.66; 0.98]
Bai et al., 2019
51
6
45
0.87
[0.76; 0.99]
Random effects model (HK)
0.79
[0.71; 0.88]
2
Heterogeneity:
I
 = 36% [0%; 78%]
EEG power
Stefan et al., 2018
39
11
28
0.68
[0.48; 0.88]
Sitt et al., 2014
71
22
49
0.71
[0.57; 0.85]
Chennu et al., 2017
61
39
22
0.76
[0.64; 0.88]
Random effects model (HK)
0.75
[0.70; 0.79]
2
Heterogeneity:
I
 = 0% [0%; 90%]
CRS-R
Stefan et al., 2018
39
11
28
0.65
[0.45; 0.85]
Chennu et al., 2017
60
38
22
0.66
[0.52; 0.80]
Bai et al., 2019
51
7
44
0.77
[0.61; 0.93]
Random effects model (HK)
0.67
[0.60; 0.75]
2
Heterogeneity:
I
 = 0% [0%; 90%]
0.4
0.5
0.6
0.7
0.8
0.9
1
