# Supplementary figures and images for: EEG connectivity and network analyses predict outcome in patients with disorders of consciousness – A systematic review and meta-analysis
Source: Heliyon. 2024 May 15;10(10):e31277. doi: 10.1016/j.heliyon.2024.e31277 (PMC11141356; doi:10.1016/j.heliyon.2024.e31277)

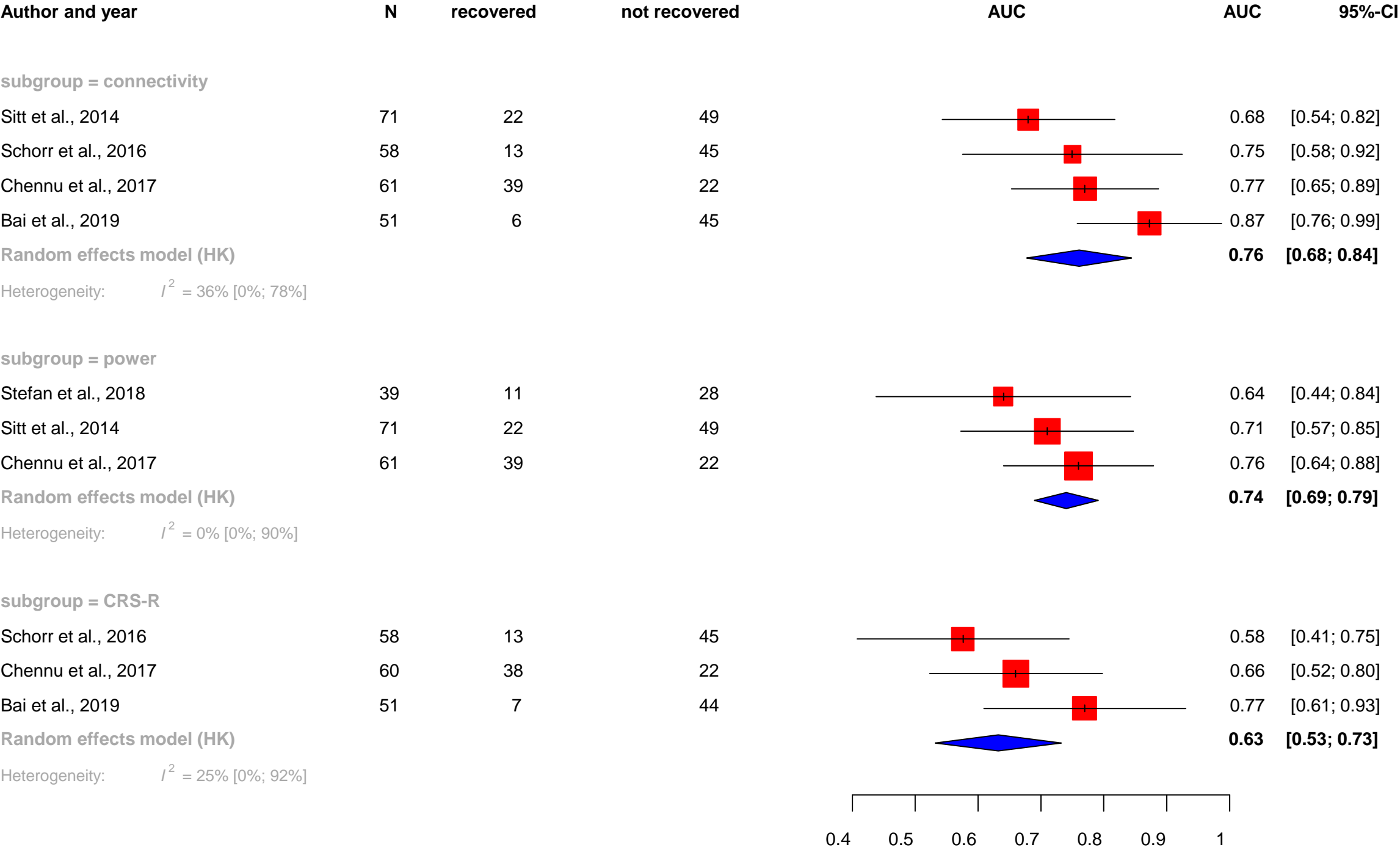

Supplement: Supplementary materials Fig. 3 — Forest-plot showing the prediction of outcome. The effect measure was the discriminative capacity (AUC) of EEG connectivity measures and network metrics, EEG spectral power and clinical scale. The red square stands for the estimates of the effect in each study, horizontal lines are the CI-s, while the blue diamond shows the estimate of the overall effect. This analysis was conducted using the data from Schorr et al. [24]. [file mmc3.pdf]
