# Supplementary material for: EEG connectivity and network analyses predict outcome in patients with disorders of consciousness – A systematic review and meta-analysis
Source: Heliyon. 2024 May 15;10(10):e31277. doi: 10.1016/j.heliyon.2024.e31277 (PMC11141356; doi:10.1016/j.heliyon.2024.e31277)
Supplement: Supplementary materials Table 1 — EEG analysis characteristics of the included articles (ICA: Independent Component Analysis, NA: Not Applicable, not mentioned in the article). [file mmc4.docx]

| **Article**  **Author, year** | **EEG analysis characteristics** | | | |
| --- | --- | --- | --- | --- |
|  | **Filtering** | **Artefact rejection** | **Epoching** | **Band definition** |
| *Chennu, 2017* | 0.5-45Hz | visual and ICA | 10 s epochs (first 60 clean epochs (i.e., 10 minutes) from each subject were retained) | delta (0–4 Hz), theta (4–8 Hz), alpha (8–13 Hz) |
| *Stefan, 2018* | high-pass filter at 0.1Hz | automatic based on voltage | NA | delta (0–4 Hz), theta (4–8 Hz), alpha  (8–13 Hz) |
| *Bai, 2019* | bandpass  filter (1–45 Hz) | ICA | 10 s epochs | delta (1–4 Hz), theta (4–8 Hz), alpha (8–13 Hz), beta (13–30 Hz), gamma  (30–45 Hz) |
| *Schorr, 2016* | 1 Hz high-pass filter and a 100 Hz  low-pass filter, 50 Hz notch filter | semi-automatic based on voltage | 2 s epochs, mean of 137 (±8) epochs per person were analysed | delta (1–4 Hz),  theta (5–8 Hz), alpha (9–13 Hz), beta (14–30 Hz), gamma (30–100 Hz) |
| *Sitt, 2014* | band-pass  filter (0.2-45 Hz) | automatic based on voltage | NA | delta (1-4 Hz), theta (4-8 Hz), alpha (8-13 Hz), beta (13-30 Hz), gamma (30-45 Hz) |
